# COVID-19-Associated Disease Course Is Shortened in Moderate-to-Severe Atopic Dermatitis Patients Receiving Dupilumab Treatment: A Retrospective Cross-Sectional Study

**DOI:** 10.3390/jcm12103415

**Published:** 2023-05-11

**Authors:** Dongxia Ma, Yin Wang, Nan Huang, Wenjing Li, Hao Chen, Yaqi Yang, Rongfei Zhu

**Affiliations:** Department of Allergy, Tongji Hospital, Tongji Medical College, Huazhong University of Science and Technology, Wuhan 430030, China; madongxia2017@163.com (D.M.);

**Keywords:** atopic dermatitis, dupilumab, COVID-19, incidence, hospitalization

## Abstract

Previous studies suggest that allergic diseases may be a protective factor in SARS-CoV-2 infection. However, data regarding the impact of dupilumab, a widely used immunomodulatory medication, on COVID-19 in an allergic population are very limited. To investigate the incidence and severity of COVID-19 among moderate-to-severe atopic dermatitis (AD) patients treated with dupilumab, a retrospective cross-sectional survey was conducted among patients with moderate-to-severe AD who presented at the Department of Allergy of Tongji Hospital from 15 January 2023 to 31 January 2023. Healthy individuals matched for gender and age were also enrolled as a control. All subjects were asked about their demographic characteristics, past medical history, COVID-19 vaccination history, and medications, as well as the presence and duration of individual COVID-19-related symptoms. A total of 159 moderate-to-severe AD patients and 198 healthy individuals were enrolled in the study. Among the AD patients, 97 patients were treated with dupilumab, and 62 patients did not receive any biologicals or systemic treatments (topical treatment group). The proportions of people who were not infected with COVID in the dupilumab treatment group, topical treatment group and healthy control group were 10.31%, 9.68% and 19.19%, respectively (*p* = 0.057). There was no significant difference in COVID-19-related symptom scores among all groups (*p* = 0.059). The hospitalization rates were 3.58% in the topical treatment group and 1.25% in the healthy control group, and no patient was hospitalized in the dupilumab treatment group (*p* = 0.163). Compared with healthy control group and topical treatment group, the dupilumab treatment group had the shortest COVID-19-associated disease duration (dupilumab treatment group, 4.15 ± 2.85 d vs. topical treatment group, 5.43 ± 3.15 d vs. healthy control group, 6.09 ± 4.29 d; *p* = 0.001). Among the AD patients treated with dupilumab for different times, there was no appreciable difference (<0.5 year group, 5 ± 3.62 d vs. 0.5–1 year group, 4.84 ± 2.58 d vs. >1 year group, 2.8 ± 1.32 d; *p* = 0.183). Dupilumab treatment shortened the duration of COVID-19 in patients with moderate-to-severe AD. AD patients can continue their dupilumab treatment during the COVID-19 pandemic.

## 1. Introduction

Coronavirus disease 2019 (COVID-19), a pandemic disease caused by SARS-CoV-2, brings about a variety of symptoms, such as a high fever, dry cough, myalgia, malaise, and olfactory and/or gustatory dysfunction and sometimes seriously endangers the health and lives of patients [1]. The pathogenesis of COVID-19 is complex. CD4^+^T and CD8^+^T cells play a significant role in defending against viral infections, during which T cells are differentiated into various subsets and activated [2,3]. It is well known that Th1 and Th17 cells are indispensable to the formation of lung inflammation. Meanwhile, massive pro-inflammatory cytokines, including interleukin (IL)-1, IL-6, IL-8, IL-21, TNF-α, and MCP-1, are released, and B cells can synthesize and secrete virus-specific antibodies [4]. However, type 2 cytokines, IL-4 and IL-13, are also a key factor in promoting and aggravating COVID-19 [5], and expressions of IL-4 and IL-13 are elevated in the serum of patients with COVID-19 [6].

The main features of atopic dermatitis (AD), a mainly chronic skin condition, include pruritus, eczematous inflammation, excoriations, scaling and dry skin [7,8]. The pathophysiology of AD is complex and multifactorial, with the imbalance of Th2/Th1 playing a vital role in the development of AD by altering cell-mediated immune responses and promoting lgE-mediated hypersensitivity [9]. As important type 2 inflammatory cytokines, IL-4 and IL-13 are crucial in the initiation, development, and exacerbation of AD [7,9]. Dupilumab, a fully human monoclonal antibody directed at the alpha subunit of IL-4 receptors, can block signal transductions downstream from both IL-4 and IL-13 [10]. Dupilumab, which is approved by the FDA for the treatment of moderate-to-severe atopic dermatitis, has been used broadly in the treatment of moderate-to-severe AD effectively and safely [11].

Preliminary reports suggest that dupilumab does not cause an elevated risk of SARS-CoV-2 infection and does not increase COVID-19 complications in patients with AD [12]. Based on the limited data, it has been suggested that the use of dupilumab should not be stopped in the treatment of moderate-to-severe AD during the COVID-19 pandemic [13]. Moreover, a recent retrospective study suggests that the overall cost-effectiveness is advantageous for dupilumab used in treating moderate-to-severe AD in the long run, although its direct cost might appear higher when compared with traditional treatment schemes [14]. In December 2022, China ended its “zero-COVID” policy, and more than 70% of the population became infected with SARS-CoV-2 within one month. The present study aims to investigate the risk of COVID-19, COVID-19-related symptom scores, COVID-19-associated symptom duration, COVID-19-associated hospitalization, and mortality among patients with moderate-to-severe AD treated with dupilumab.

## 2. Materials and Methods

### 2.1. Study Design and Subjects

The survey was conducted among patients with moderate-to-severe AD who presented at the Department of Allergy of Tongji Hospital from 15 January 2023 to 31 January 2023. The diagnosis of AD was made according to the Hanifin and Rajka criteria [15], and the total lgE (TlgE) test was conducted and analyzed for all AD patients. AD was further classified into extrinsic and intrinsic phenotypes, primarily according to TlgE levels [16]; namely, those with TlgE values of 200 kU/L and higher belonged to the extrinsic AD category, and the rest, with TlgE values of less than 200 kU/L, belonged to the intrinsic AD group. For the dupilumab treatment group, those aged 18 and above received 600 mg of dupilumab as the loading dose, and they then received 300 mg every 2 weeks. Meanwhile, for the AD patients under 18, the dose administered varied according to body weight. Patients weighing 60 kg or more received 300 mg of dupilumab every 2 weeks, with dupilumab 600 mg as the loading dose; patients weighing below 60 kg received 300 mg of dupilumab every 4 weeks without a higher loading dose. Moreover, the patients needed to fulfill the following criteria: (1) they were treated with dupilumab within 61 days prior to their first COVID-19 diagnosis date [17]; (2) they did not receive any other biological agent or systemic treatment. For the topical treatment group, the patient should not have received any biologicals or systemic treatments. Healthy controls matched with the AD patients for gender and age were also enrolled for the same study period and were identified through advertising. The healthy controls came from local schools, hospital employees and from the general population in Wuhan. The healthy controls were participants free of diseases according to their medical history, and they usually did not take any medicine. This study was approved by the Independent Ethical Committee of Tongji Hospital (NO. TJ-IRB20230204). All the subjects or their guardians provided signed written informed consent.

All the subjects were asked about their past medical histories, COVID-19 vaccination histories, medications, and demographics (i.e., age, gender, and BMI), as well as the presence and duration of individual COVID-19-related symptoms, including objective or subjective fever, sore throat, cough, nasal congestion, runny nose, nasal itch, sneeze, headache, fatigue, anosmia, dysgeusia, nausea, vomiting, diarrhea, anorexia, skin changes, hospitalization status, nucleic acid or antigen test report, and SARS-CoV-2 infection exposure.

All participators were diagnosed with COVID-19 infection according to PCR testing for nucleic acid, SARS-CoV-2 antigen testing, and the presence of positive symptoms and a high-risk COVID-19 exposure, such as through family, co-workers, or colleagues with a documented COVID-19 infection. The duration of COVID-19 was calculated from the day on which the patients felt uncomfortable with any of the symptoms above to the time at which they recovered from the illness and were without any clear discomfort. The incidence of COVID-19 and the duration of COVID-19-related symptoms were compared in all groups. In addition, in the study, the severity of the symptoms was also analyzed based on the methods reported by Benjamin Ungar et al. [18]. In brief, there were five levels of scores from 0 to 5, i.e., 0 = asymptomatic; 1 = mild disease (the patients were without fever and dyspnea, and the disease resolved in <7 days; in short, it resembled a common cold); 2 = moderate disease (some fever and/or cough, or with other lower respiratory symptoms, which resolved at home in 7–14 days); 3 = severe disease (these patients contracted pneumonia and required hospitalization, but they resolved without intubation); 4–5 = very severe disease (the patients required hospitalization, intubation, and even other supportive measures) or fatal.

### 2.2. Statistical Analyses

Data were analyzed using SPSS 22.0 software. Descriptive parameters such as means and standard deviations were calculated for normally distributed continuous data, and frequencies and percentages were calculated for categorical data. Pearson’s χ^2^ test and Fisher’s exact test were used to determine the correlations between the categorical variables. A one-way ANOVA was used to evaluate the continuous variables. The comparisons among groups were performed with the LSD test or Tamhane’s T2 test. Continuous variables with unequal variances were evaluated using the Kruskal–Wallis H test.

## 3. Results

### 3.1. Characteristics of the Study Population

A total of 159 patients with moderate-to-severe AD were enrolled in the study. They were divided into two groups: 97 were treated with dupilumab (dupilumab treatment group), and 62 did not receive any biological agent or systemic treatment (topical treatment group). Moreover, in AD treatment, no JAK inhibitor or any other biological therapies were used in all moderate-to-severe AD patients. There was no significant difference in the duration of AD between the dupilumab treatment group and topical treatment group. The AD phenotype was divided into an extrinsic status and an intrinsic status based mainly on the TlgE level, and there was no statistical difference in the extrinsic and intrinsic status composition between the dupilumab treatment group and the topical treatment group (TlgE level: dupilumab-treatment group, 875.77 ± 1116.76 KU/L vs. topical-treatment group, 1005.05 ± 1442.82 KU/L, *p* = 0.942; AD phenotype, dupilumab-treated group vs. topical treatment group, *p* = 0.550). The status analysis of atopic dermatitis is shown in Table 1.

Meanwhile, after excluding individuals with any disease, we also recruited 198 healthy controls for the survey. Age and gender were well matched among all groups. The 2019-nCoV vaccine administered to all individuals in Wuhan was inactivated. The proportion of patients receiving the COVID-19 vaccine did not differ significantly among all three groups (*p* = 0.825). However, participants in the healthy control group received more doses of the 2019-nCoV vaccine compared with those in the dupilumab treatment group and topical treatment group (*p* = 0.001 and *p* = 0.034, respectively; shown in Table 2), although there was no significant difference in doses per person between the dupilumab treatment group and the topical treatment group (*p* = 0.904). Demographics and comorbidities are listed in Table 2. To reduce the bias induced by age, we further compared the age composition among all groups. The proportion of young patients (≤17 years) was similar among all three groups (*p* = 0.651), and there was no significant difference in ages (dupilumab treatment group, 7.92 ± 4.08 y vs. topical treatment group, 7.64 ± 3.26 y vs. healthy control group, 8.24 ± 4.37 d; *p* = 0.721).

### 3.2. COVID-19 Severity Analysis

**Figure 1:**
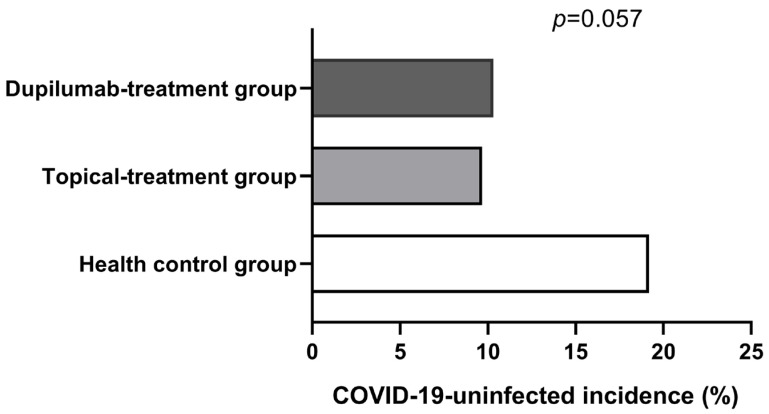
Comparison of COVID-19-uninfected incidence among all groups. COVID-19-uninfected incidences were 19.19%, 9.68% and 10.31% in Health control group (white columnar), Topical-treatment group (grey columnar) and Dupilumab -treatment group (black group) respectively.

**Figure 2:**
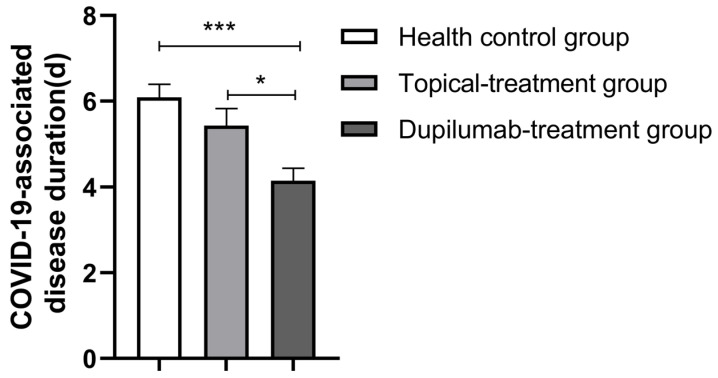
Comparison of COVID-19-associated disease duration in the healthy control group, topical treatment group and dupilumab treatment group (mean ± SEM, * *p* < 0.05, *** *p* < 0.001).

**Figure 3:**
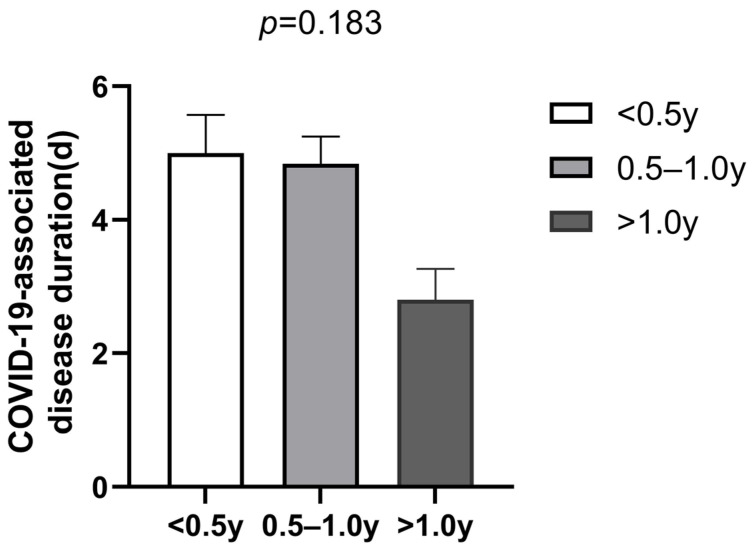
Comparison of COVID-19-associated disease duration in dupilumab treatment group for the <0.5 year, 0.5–1.0 year and >1.0 year subgroups (mean ± SEM, *p* value generated from comparison of all three groups was 0.183 with Kruskal–Wallis H test).

**Figure 4:**
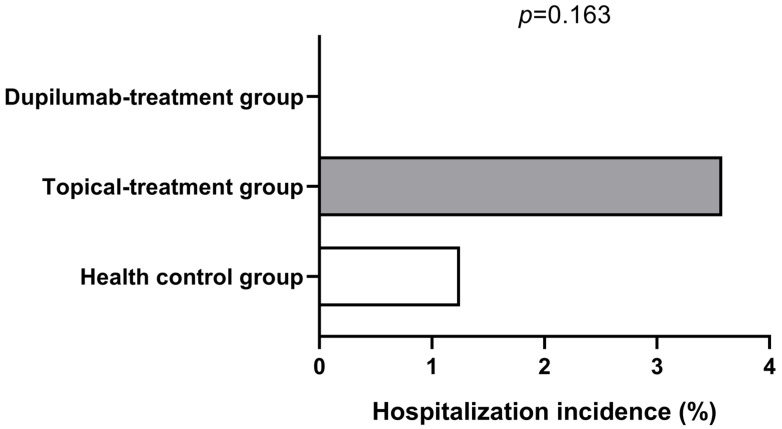
Comparison of hospitalization incidence in the healthy control group (1.25%, white columnar), topical treatment group (3.58%, grey columnar), and dupilumab treatment group (0%, the *p*-value generated from the comparison of all three groups was 0.163 with Pearson’s χ^2^ test and Fisher’s exact test).

All participants were diagnosed with COVID-19 infection based on PCR testing to detect nucleic acid or/and SARS-CoV-2 antigen testing with elevated values and the presence of positive symptoms and high-risk exposure to COVID-19. As is shown in Figure 1, the COVID-19 infection rate was very high among all groups. Although the AD patients in the topical treatment group had a higher COVID-19 infection rate compared with the AD patients in the dupilumab group and patients in the healthy control group, there was no statistical difference among the three groups (*p* > 0.05). However, we found that the dupilumab treatment group had the shortest COVID-19-associated disease duration compared with healthy control group and the topical treatment group (dupilumab treatment group, 4.15 ± 2.85 d vs. topical treatment group, 5.43 ± 3.15 d vs. healthy control group, 6.09 ± 4.29 d; *p* = 0.001; shown in Figure 2), yet the COVID-19 duration was similar between the topical treatment group and the healthy controls (*p* > 0.05). In addition, no appreciable difference was found after we compared the COVID-19 duration of AD patients treated with dupilumab for different amounts of time (<0.5 year group, 5 ± 3.62 d vs. 0.5–1 year group, 4.84 ± 2.58 d vs. >1 year group, 2.8 ± 1.32 d; *p* = 0.183; as is shown in Figure 3). The hospitalization rates were very low in all groups, and there was no significant difference among all three groups in the incidence of COVID-19-associated hospitalization (shown in Figure 4). In this study, there was no occurrence of COVID-19-associated mortality.

**Figure 5:**
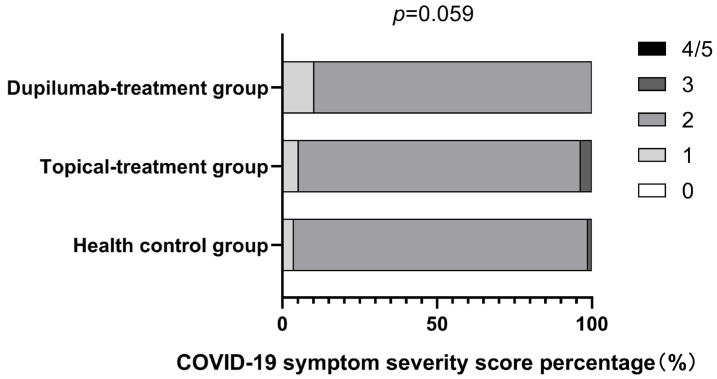
COVID-19 symptom severity score in all individuals with COVID-19 infection in each group (*p* value generated from comparison of all three groups was 0.059 with the Kruskal–Wallis H test).

Furthermore, the severity of COVID-19-related symptoms was compared and is shown in Figure 5. There were no individuals with COVID-19 symptom severity scores of “0” and “4–5” in all groups among the patients infected with COVID-19. It is noticeable that the proportion of patients with a symptom score of “2” was the highest among all three groups, and this was followed by the proportion of patients with a symptom score of “1”. The proportion of patients with a symptom score of “3” was rather low (dupilumab treatment group, 0% vs. topical treatment group, 3.57% vs. healthy control group, 1.25%). Overall, there was no significant difference in COVID-19-related symptom scores among all groups (*p* = 0.059).

**Figure 6:**
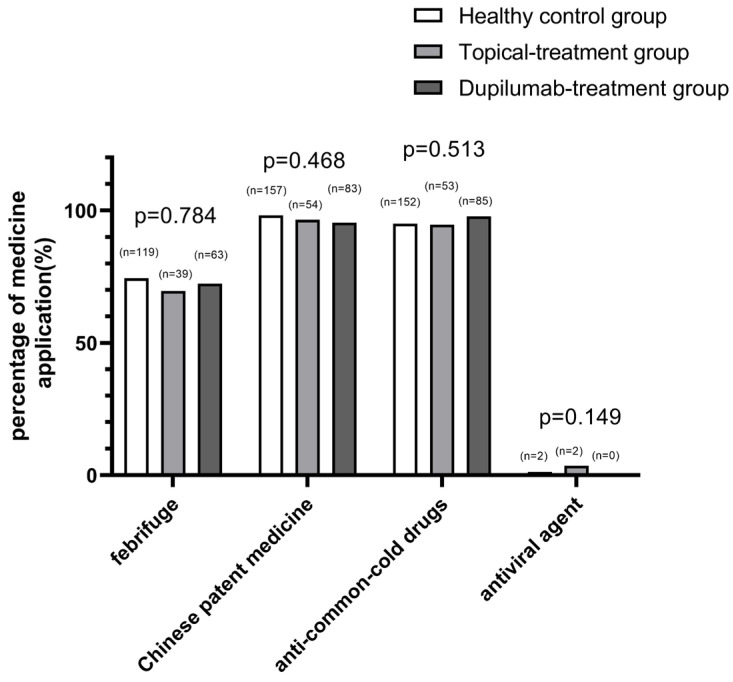
Comparison of the application of medicines for COVID-19 treatment in the healthy control group, topical treatment group and dupilumab treatment group (*p*-values generated from a comparison of all three groups were 0.784, 0.468, 0.513 and 0.149, respectively, for the use of febrifuge, Chinese patient medicine, anti-common-cold drugs, and an antiviral agent, determined with Pearson’s χ^2^ test and Fisher’s exact test; n represents the number of individuals in each group using the corresponding medicine).

Meanwhile, we investigated and analyzed the drugs used for the treatment of COVID-19 in each group. In December 2022, when the “zero-COVID” policy ended in China, about 70% of the population became infected with SARS-CoV-2. Concurrently, the country formulated guidelines for the use of drugs in the fight against COVID-19, and people were treated for COVID-19 according to the guidelines. There was no significant difference in the application of medicine, including febrifuge (*p* = 0.784), Chinese patient medicine (*p* = 0.468), anti-common-cold drugs (*p* = 0.531), and an antiviral agent (*p* = 0.149), among all groups (shown in Figure 6). Although there may be some differences in the specific names of medications, the types of medications were basically the same.

## 4. Discussion

COVID-19 is a pandemic that has been affecting the world’s population for three years. Many factors influence susceptibility to COVID-19 [19,20]. Current reports have revealed controversial findings for the connection between AD and COVID-19 occurrence. Ryan Fan et al. reported that AD is associated with a higher incidence of COVID-19 occurrence [21]. Meanwhile, some studies reported that allergic rhinitis, AD and other atopic conditions are protective factors against COVID-19 infection for the following reasons [22,23]. Firstly, allergic diseases are mainly characterized by type 2 inflammation. IL-4 and IL-13, as important cytokines of the Th2 immune response, negatively regulate the expression of angiotensin-converting enzyme II (ACE2) on the airway epithelial cell through which SARS-CoV-2 enters the host cell [24]. Secondly, eosinophils play a protective role against SARS-CoV-2 infection. An eosinophil count reduction was usually observed in COVID-19 patients, and it is more prominent in patients with severe COVID-19 than in patients with mild COVID-19 [25]. Furthermore, SARS-CoV-2 infection downregulates CRTH2 (CD294), a high-affinity receptor of prostaglandin D, which is a central activator of the type 2 inflammation response and is expressed in eosinophils [25]. Thus, it further decreases ACE2 expression. Our study is consistent with the most studies that see AD as a likely protective factor; at least, it is not a risk factor for SARS-CoV-2 infection. In the study, we found that AD patients receiving topical treatment were not more likely to become infected with COVID-19, although the proportion of people who were not infected with COVID-19 in the topical treatment group was slightly lower (*p* = 0.057), which may be explained by the different vaccine doses for individuals as the number of vaccine doses per person increased significantly in the health control group compared with the topical treatment group and dupilumab treatment group. 

The interaction between SARS-CoV-2 and the aberrant immune system in AD patients is not well elucidated. AD is a common inflammatory skin disease, and its immunopathological mechanism is complex. Both genetic and environmental factors are involved in the occurrence and development of AD that synergistically drive immune imbalance, resulting in a variety of immune cells, such as Th1/Th2/Th17/Th22 cells and an abnormal immune response in the skin tissue [26]. These immune cells play a different role during acute and chronic phases of AD. The acute phase is characterized by the activation of Th2/Th22 cells, and in the chronic phase, although it is characterized by a marked Th 1 polarization, Th2 cells also play an important role. IL-4 and IL-13, as major type 2 inflammatory cytokines, are involved in AD. Interestingly, COVID-19 pathogenesis bears some resemblance to that of AD. SARS-CoV-2 infection can cause an incongruous immune response of innate and acquired immunity, and an imbalance of Th1/Th2 plays a significant role in the pathophysiology of COVID-19. Th2 responses facilitate inflammation and lung damage in COVID-19 patients [5]. SARS-CoV-2 spike proteins that weigh less than 70 kDa can activate Th2 cells, generating a great deal of IL-4 and IL-13, which are elevated in both mild and severe COVID-19 patients [6]. Moreover, IL-4 and IL-13 trigger the reactions of Th2 cells with positive feedback loops. Alexandra Donlan et al. demonstrated that IL-13 aggravates the COVID-19 condition by promoting the deposition of hyaluronic acid in the lungs, resulting in the need for mechanical ventilation in patients with elevated levels of IL-13 [17]. The increased levels and potential pathogenic effects of IL-4 and IL-13 in AD and COVID-19 suggest that blockades against IL-4/13 may be of some benefit for AD patients infected with SARS-CoV-2.

Dupilumab, a fully human monoclonal lgG4 antibody targeting the alpha subunit of IL-4 receptor, is an effective therapy for moderate-to-severe AD. A phase IIa trial demonstrated that dupilumab reduces the probability of admission to the intensive care unit for patients with moderate to severe COVID-19 and improves the survival rate of patients by disabling the signal transduction of IL-4 and IL-13 [27]. Patients with moderate-to-severe AD treated with dupilumab are less likely to experience severe COVID-19 versus those without dupilumab and systemic drug therapy [18]. In the present study, we found that in comparison with the healthy control group and topical treatment group, moderate-to-severe AD patients treated with dupilumab had the shortest COVID-19-associated disease course, although *p*-value (0.046) from the dupilumab treatment group compared with the topical treatment group was close to the limit of significance, which may be explained by the small sample size. In this study, we could not draw the conclusion that dupilumab treatment remarkably improves COVID-19 symptom severity score because the *p*-value (0.059) was borderline significant, probably, also due to the small sample size. Although the proportion of those who were not infected with COVID-19 in the dupilumab treatment group was slightly lower (*p* = 0.057) in this study, it may be explained by lower number of vaccine doses per person in the dupilumab treatment group compared with the healthy control group. The potential pathway through which dupilumab helps patients recover from COVID-19 might be that dupilumab restricts the duplication of SARS-CoV-2 and alleviates the abnormal immune responses in AD patients, possibly by blocking the augmented reaction caused by IL-4 and IL-13.

Moreover, in this survey, we also found that in the dupilumab treatment group, the COVID-19 course was not related to the duration of dupilumab treatment, the reason being that there was no appreciable difference among the subgroups treated with dupilumab for less than 0.5 year, 0.5–1.0 year, and more than 1.0 year, which might be attributed to the quick suppression of type 2 inflammation by dupilumab without an additive effect. It has been demonstrated that dupilumab has some effect on eosinophils, which are important innate immune cells [28]. Eosinophils have been shown to have an antiviral activity due to the expression of endosomal toll-like receptors, eosinophil-derived neurotoxin (EDN/RNAse2), eosinophil cationic protein (ECP/RNAse3) and inducible NO synthase [29]. Many studies suggest the eosinophil count as an indicator of COVID-19 severity, for example, asthmatics with an increased peripheral blood eosinophil count generally have a more favorable outcome [30,31]. Previous studies in AD patients suggest that dupilumab treatment may induce transient increases in mean eosinophil counts and, especially in the first 4 weeks, this increases significantly [28]. Therefore, we hypothesize that dupilumab may alleviate COVID-19 to some extent, especially in the early stage. Nevertheless, in the cross-sectional study, we did not analyze eosinophil counts in all groups and did not compare eosinophil count changes in the AD patients treated with dupilumab.

Thus, more fundamental data are needed to validate our findings. This study also indicated that hospitalization rates were very low in all groups, and in particular, no patient was hospitalized in the dupilumab treatment group, which further implied the potential protective effect of dupilumab in COVID-19 patients, although no significant difference was found among the three groups. In addition, the very low hospitalization rate and lack of death cases in this study may be attributed to the SARS-CoV-2 variants having more remarkable immune escape abilities and thus a reduced virulence/lethality than other variants seen before.

It is well known that vaccination prevents infection with SARS-CoV-2 and severe COVID-19 to some degree. Additionally, the protection efficacy varies by doses. In this study, there were no significant differences in vaccination rates among the three groups; however, compared with the topical treatment group and dupilumab treatment group, the vaccine doses per person were significantly higher in the health control group. The possible reason for this is because the moderate-to-severe AD patients, especially the patients who developed allergic reactions after the first vaccination, were more worried about allergic reactions to vaccination, and they were likely to refuse another COVID-19 vaccination. The vaccine doses per person in the dupilumab treatment group and topical treatment group appeared similar (*p* = 0.747). The above factors may explain why the proportion of those who were not infected with COVID-19 in the health control group is slightly higher (dupilumab treatment group, 10.31% vs. topical treatment group, 9.68% vs. healthy control group, 19.19%; *p* = 0.057), although the *p*-value was borderline significant. Furthermore, the protection efficacy is also related to dosing interval. However, it is very difficult to measure the specific impact. Therefore, inevitably, it brought about some bias in this study.

It has been demonstrated that COVID-19 is generally mild in children, although it may be severe in those with certain comorbidities. Therefore, in order to reduce the bias caused by age, we further compared the young proportion and young mean age among all groups. There was no statistical difference in both the young proportion and the young mean age (*p* = 0.651 and *p* = 0.721, respectively).

There are some other limitations to this study. First, this was a single-center study, and the sample size was relatively small, which might lead to sampling bias. Additionally, sampling bias was also inevitable in the subgroups that were treated with dupilumab for different amounts of time. Second, the patients needed to recall their COVID-19 symptoms; thus, recall bias was inevitable. Third, the treatments of COVID-19 were not compared in detail in the study, although the treatment regimens were similar in the majority of the subjects, according to the guidelines formulated by our country for the use of drugs in the fight against COVID-19. Finally, in all groups in our study, the population was relatively young; thus, we are not sure if the following conclusions can be extrapolated to the elderly.

## 5. Conclusions

In conclusion, the small sample study suggests that dupilumab treatment can shorten the duration of COVID-19 in patients with moderate-to-severe AD. AD patients do not need to discontinue dupilumab treatment during the COVID-19 pandemic.

## Figures and Tables

**Table 1:** Status analysis of atopic dermatitis in dupilumab treatment group and topical treatment group.

Status of AD	Topical treatment Group (n = 62)	Dupilumab-Treatment Group (n = 97)	*p*-Value
Duration of AD, mean (SD), years	5.85 (4.63)	5.65 (5.35)	0.728
TlgE (KU/L)	1005.05 (1442.82)	875.77 (1116.76)	0.942
Phenotypes			0.550
Intrinsic (No)	22	30	
Extrinsic (No)	40	67	

**Table 2:** Descriptive characteristics of the patients in all three groups.

Characteristic	Health Control Group (n = 198)	Topical Treatment Group (n = 62)	Dupilumab- Treatment Group (n = 97)	*p*-Value
Age, mean (SD), years	17.67 (13.11)	18.92 (14.59)	18.86 (17.05)	0.603
≤17 years old number (percent)	120 (60.61)	36 (58.06)	63 (64.95)	0.651
≤17 years old mean (SD), years	8.24 (4.37)	7.64 (3.26)	7.92 (4.08)	0.721
Gender				0.825
male	114 (57.58%)	33 (53.22%)	54 (55.67%)	
female	84 (56.58%)	29 (46.78%)	43 (44.33%)	
COVID-19 vaccinated number (percent)	172 (86.87%)	49 (79.03%)	71 (73.20%)	0.825
COVID-19 vaccine doses for individuals	2.13 (0.97) ^#^	1.76 (1.00) ^##^	1.65 (1.14) ^###^	0.000
With other disorders				
No		22 (35.48%)	31 (31.96%)	0.646
Allergic rhinitis		31 (50%)	49 (50.52%)	0.949
Nasosinusitis		8 (12.9%)	9 (9.28 %)	0.471
Food allergy		16 (25.81%)	34 (35.05%)	0.221
Drug allergy		6 (9.68%)	3 (3.09%)	0.161
Obesity		1 (1.61%)	4 (4.12%)	0.675
Cardiovascular and cerebrovascular diseases		1 (1.61%)	2 (2.06%)	1.00
Kidney disease		3 (4.84%)	2 (2.06%)	0.608
Diabetes mellitus		0 (0%)	1 (1.03%)	1.00
Tumor		1 (1.61%)	1 (1.03%)	1.00

^#^ Topical treatment group compared with health control group, *p* = 0.003; ^##^ dupilumab treatment group compared with health control group, *p* = 0.001; ^###^ dupilumab treatment group compared with topical treatment group, *p* = 0.747.

## Data Availability

Not applicable.
